# Knowledge, attitudes, and factors associated with vegetarianism in the Saudi Population

**DOI:** 10.1186/s12889-023-15636-5

**Published:** 2023-04-12

**Authors:** Wedad Azhar, Sanaa Aljabiri, Taqwa Bushnaq, Firas S. Azzeh, Reema A. Alyamani, Sarah O. Alkholy, Walaa E. Alhassani, Wafaa F. Abusudah, Alaa Qadhi, Hassan M. Bukhari, Elsayed H. Bakr, Khloud Ghafouri

**Affiliations:** 1 Clinical Nutrition Department, Faculty of Applied Medical Sciences, Umm Al-Qura University, Makkah, Saudi Arabia; 2 Department of Food Science and Nutrition, College of Sciences, Taif University, Taif, Saudi Arabia

**Keywords:** Vegetarianism, Vegetarian Diet, Vegetarian products, Dietary requirement, Vegan, Saudi Arabia

## Abstract

**Background:**

In recent years, there has been great interest in the dietary practices of vegetarians in addition to an increasing awareness of the potential benefits of vegetarian diets. However, there are insufficient data on the spread of vegetarianism in Arab countries. The aim of this study was to investigate knowledge and attitudes about vegetarianism and associated factors in the Saudi population and to understand the reasons for its growing prevalence.

**Method:**

This is a cross-sectional study began in May 2020 and ended in September 2020. Researchers distributed the questionnaire electronically through social media. Data were collected electronically and exported to Excel by the researchers .The electronic questionnaire comprised three sections: sociodemographic questions, reasons for following a vegetarian diet, and beliefs and knowledge about vegetarianism.

**Results:**

There were 3,035 responses, of which 80.2% of respondents were female and 19.8% were male. Participant were aged 18–65. Vegetarians represented 37.5% (15.7% semivegetarians, 8.1% pescovegetarians, 64.3% lacto-ovo-vegetarians, and 11.3% vegans), and the rest were nonvegetarians. The majority of the vegetarian participants (92.9%) had a low vegetarianism knowledge level. Health issues, followed by ethical and environmental concerns, were the most common motivators for adopting a vegetarian diet; these reasons were cited by 72.5%, 59.3%, and 47.9% of participants, respectively. Factors associated with increased vegetarianism were engaging in exercise for half an hour to two hours, while factors associated with decreased vegetarianism were male, aged 51–64 years, being married, having a higher education, working in the health sector, being a housewife, and having an income between 5,000 and 10,000 SR/month.

**Conclusion:**

Vegetarianism appears to be a growing phenomenon among the Saudi population. Increased awareness of health issues and the desire to live a healthy lifestyle might be the strongest motives. This study offers an opportunity to better understand vegetarianism in Saudi Arabia along with the possibility of expanding vegetarian food choices for the general public.

## Background

Food choice is a complex behaviour based on many interacting variables related to the individual, the product, and the context [1]. There are many physiological, psychological, biological, genetic and sociocultural factors that influence an individual’s food choices that should be considered when studying a specific eating behaviour [2]. Although the current trend in Europe involves consumption of a Western diet [3], which is associated with the consumption of animal products, refined carbohydrates, and added sugars, vegetarianism is becoming more popular than before [4, 5, 6]. Vegetarianism is a general term that refers to refraining from consuming one or more types of foods from animal sources; however, many variations exist [7]. Vegetarians can be subclassified into lacto-ovo-vegetarians (who consume eggs and dairy but no meat, poultry, fish, or seafood), ovo-vegetarians (who consume eggs but not dairy), lacto-vegetarians (who consume dairy products but not eggs), pesco-vegetarians (who consume fish and seafood but not meat and poultry), and pollo-vegetarians (who consume poultry but not meat and fish) [8].

In recent years, there has been global interest in the potential benefits of dietary practices of vegetarians, both in terms of nutritional content and implications for overall health and mortality rates [9].

The number of vegetarians in Saudi Arabia is unknown; however, it has been estimated in some countries, such as 8% in Canada [10], 5% in the US [11], 4.3% in Germany [12], 8% in South Australia [13], 15% in Scandinavia [14], less than 2% in France [15], 6% in the UK [16], 1–2% in Argentina [17], 33% in South Asia [18], and 40% in India [19]. The insufficient data on the spread of vegetarianism might be because there is no widely accepted definition of vegetarianism [20].

There are many reasons why people follow a vegetarian diet: ethical reasons, such as animal welfare; health concerns (including weight loss); economic reasons; social reasons (such as the influence of family and friends); sensory preferences and taste; food safety concerns; and religious beliefs [4, 17, 20]. Dislike of meat may be another reason for choosing a vegetarian lifestyle [21]. Recent Western studies indicate that ethical reasons are the most common motivation for adopting a vegetarian diet, followed by health concerns and then environmental concerns [21–24] Click or tap here to enter text. The practice of vegetarianism in India tends to be based on religious and traditional reasons rather than health concerns. Therefore, India ranks first among countries with the largest number of vegetarians, estimated at 300–400 million [19]. The dietary habits of Saudis shift towards animal-origin foods. However, consuming plant-origin foods for Saudis was favoured by those who wanted to reduce their bodyweight and have better health, those who were concerned with animal rights, and/or those with certain religious beliefs [24]. To our knowledge, the prevalence of vegetarians, attitudes towards vegetarianism and motivations for vegetarianism have not yet been studied in Saudi Arabia or the Gulf countries. Therefore, the aim of this study is to investigate knowledge and attitudes about vegetarianism and associated factors in the Saudi population and to understand the reasons for its growing prevalence.

## Methodology

### Participants

This cross-sectional study began in May 2020 and ended in September 2020. Any pregnant or lactating woman and any participant with chronic disease(s) were excluded from the study. Participants were recruited randomly through social media platforms, specifically Facebook, Twitter and LinkedIn, by posting the study link in all researchers’ personal pages, the official page of the college, and the profiles of two social media influencers who agreed to help. When posting the link, researchers used the most popular hashtags on each website. Additionally, participants were recruited through WhatsApp groups (e.g., student groups, coworker groups), and at the end of each message, researchers asked recipients to forward the link to everyone’s contacts. This practice is widely used in Saudi Arabia for viral marketing.

### Sample size

The Raosoft sample size calculator (http://www.raosoft.com/samplesize.html) was used to calculate the sample size needed for this study. The total population of Saudi Arabia is approximately 30 million citizens. The predetermined margin of error was 5%, and the confidence level was 95%. The target sample size was 385 to minimize erroneous results and increase study reliability.

### Questionnaire

The questionnaire was released on SurveyHero. All questionnaire results were downloaded from the website for analysis. Data were then exported by the researchers to Excel in order to prepare the data to statistical analysis. The questionnaire was written in both Arabic and English. Participation in the survey was voluntary. The introduction to the questionnaire included an explanation of the purpose of the research and a consent form. It was also mentioned that the time needed to complete all the questions was approximately 10 min. The questionnaire contained three sections. The first section was about sociodemographic data, including sex, age, place of residency, marital status, educational level, job, income, smoking habits, presence of chronic diseases, and exercise frequency. The next question asked whether the participant was a vegetarian or a nonvegetarian. The participants were also asked about the reasons that led them to choose a vegetarian diet and the obstacles to adhering to this diet. BMI was calculated for all participants. Their nutritional knowledge and attitude were also assessed through a group of Likert scale statements. To assess participants’ knowledge, four questions were asked. Each correct answer was given one point, and the total sum of the discrete scores of the different items was calculated. Participants who correctly answered two questions or less were classified as having a “low knowledge level”, while participants who correctly answered more than two questions were classified as having a “moderate to high knowledge level”. Restrictions were put in place to ensure that participants completed the survey only once. Incomplete answers and answers that were deemed to be typos or were clearly false were excluded.

### Pilot study

To ensure the reliability and validity of the study questions, the questionnaire was designed by a third party who had experience creating qualitative questionnaires. The questionnaire was based on previous studies [23–26] and modified to accommodate the Saudi population. The dietary components included in the questionnaire were evaluated by a nutritionist. A pilot study on 30 participants was conducted to validate the questionnaire and test the questionnaire under survey conditions, in addition to determining the duration of the interview.

The study was performed in accordance with the Declaration of Helsinki and approved by the Biomedical Ethics Committee at Umm Al-Qura University (HAPO-02-K-012-2020-10-447). All participants signed informed consent forms prior to participating. Data were stored in secure computer in files that have password, protected and encrypted. Only the researchers and statistician have access to the data.

### Statistics

Data analysis was performed using Statistical Package for the Social Sciences (SPSS) (IBM SPSS Statistics for Windows, Version 23.0. Armonk, NY: IBM Corp). Frequencies and rates were used to present categorical variables. The chi-square test was used to test for correlations between categorical variables. Multivariate binary logistic regression was utilized to determine predictive factors for being a vegetarian, and the following variables were entered into the model: sex, age, marital status, educational level, job, income, smoking habits, presence of chronic diseases, and exercise frequency. The model’s goodness-of-fit was tested using the Omnibus test and the Hosmer and Lemeshow test. The level of significance was set at *P* ≤ 0.05. Cronbach’s alpha for all tested knowledge and attitude domains was greater than 0.7, indicating acceptable reliability.

## Results

### Participants

Out of 3,265 responses collected, 230 were excluded because they were incomplete. There were 3,035 completed responses (response rate equals 93%). Participants were residents of Saudi Arabia aged 18–65 years. Participants were Female and male respondents numbered 2,434 (80.2%) and 601 (19.8%), respectively. The respondents were from all regions of the Kingdom (Table 1).

**Table 1:** Socio-Demographic Profile of the Participants (N = 3,035)

Demographical Characteristics	N (%)
**Gender**	
Female	2,434 (80.2)
Male	601 (19.8)
**Age**	
18–39	2,772 (91.3)
40–50	192 (6.3)
51–64	65 (2.1)
65 years and older	6 (0.2)
**Nationality**	
Saudi	2,852 (94)
Non-Saudi	183 (6)
**Place of Residency**	
Makkah Region	1,099 (36.2)
Madinah Region	220 (7.2)
Riyadh Region	754 (24.8)
Qasim Region	162 (5.3)
Baha Region	100 (3.3)
Tabuk Region	47 (1.5)
Northern Borders Region	53 (1.7)
Eastern Region	410 (13.5)
Jouf Region	30 (1)
Jazan Region	50 (1.6)
Najran Region	14 (0.5)
Other	96 (3.2)
**Marital Status**	
Single	2,127 (70.1)
Engaged	72 (2.4)
Married	743 (24.5)
Widowed	10 (0.3)
Divorced	71 (2.3)
Separated	12 (0.4)
**Educational Level**	
Intermediate or less	57 (1.9)
High school	813 (26.8)
Diploma	161 (5.3)
Bachelor’s degree	1,686 (55.6)
Higher Education	318 (10.5)
**Occupation**	
In the public sector	263 (8.7)
In the health sector	274 (9)
In the health sector but not a health practitioner	42 (1.4)
In the private sector	287 (9.5)
In the army / police	44 (1.4)
Self-employed	91 (3)
Unemployed	774 (25.5)
Housewife	163 (5.4)
Student	1053 (34.7)
Retired	31 (1)
Unable to work	13 (0.4)
**Income**	
Less than 5,000 SR/month	2095 (69)
5,000–10,000 SR/month	419 (13.8)
10,000–15,000 SR/month	264 (8.7)
15,000–20,000 SR/Month	122 (4)
More than 20,000 SR/month	135 (4.4)

### Medical history of participants and lifestyle

Overall, the majority of the participants (59.1%) did not suffer from any disease, whereas 12% had gastrointestinal diseases, 10.2% had anaemia, 7.8% had depression, 7.8% had anxiety, 6.6% had pulmonary diseases, 5.4% had food intolerance, 3% had thyroid disorders, 2.3% had cardiovascular disease and hypertension, 2.2% had diabetes, 1.9% had an abnormal lipid profile, 1.6% had immune diseases, 0.7% had gout, 0.5% had skin diseases, 0.2% had favism disease, 0.2% had epilepsy, and 1.4% had other diseases. The lifestyles of the participants are shown in Table 2.

**Table 2:** Participants’ lifestyle

	N (%)
**BMI**	
Underweight	195 (6.4)
Normal	1,751 (57.7)
Overweight	638 (21)
Obese 1	256 (8.4)
Obese 2	132 (4.3)
Obese 3	63 (2.1)
**Smoking Habits**	
Smokers	257 (8.5)
Nonsmokers	2,534 (83.5)
Occasional Smokers	244 (8)
**Exercise**	
Do not exercise	1,256 (41.4)
Half an hour/day	929 (30.6)
An hour/day	634 (20.9)
Two hours/day	173 (5.7)
More than two hours/day	43 (1.4)

### Prevalence of vegetarianism among participants

The total number of participants who self-identified as vegetarians was 1,197, of whom 188 (15.7%) were semivegetarians, 98 (8.1%) were pesco-vegetarians, 777 (64.3%) were lacto-ovo-vegetarians, and 134 (11.3%) were vegans. The number of nonvegetarians was 1,898 (62.5%). Regarding the duration of following a vegetarian diet, 117 (9.8%) of the participants said they had done so for a month or less, 162 (13.5%) for six months or less, 176 (14.7%) for a year or less, 570 (47.6%) for one to five years, and 172 (14.4%) for more than five years.

### Reasons for following a vegetarian Diet

Table 3 explains the reasons for following a vegetarian diet. Health reasons, followed by ethical reasons, and then environmental concerns were the most common motivators to adopt a vegetarian diet; these reasons were cited by 72.5%, 59.3%, and 47.9% of participants, respectively. Other reasons for following a vegetarian diet were as follows: do not like to eat meat (33.7%), meats are injected with hormones, and that causes diseases (36.9%), to enhance mood (18.5%), weight loss and spiritual reasons (16.2%), to get rid of acne (13.7%), and psychological reasons (12.9%).

**Table 3:** Reasons for Following a Vegetarian Diet (N = 1,197)

Question	N	%
Because I think a vegetarian diet is healthier	868	72.5
Because I care about animal rights	710	59.3
Because I want to preserve the ecosystem by reducing animal farming that increases the problem of global warming	573	47.9
I do not like to eat meat	403	33.7
Meats are injected with hormones, and that causes diseases	442	36.9
To enhance mood	221	18.5
Weight loss	194	16.2
Spiritual reasons	194	16.2
To get rid of acne	164	13.7
Psychological reasons (trauma related to animal slaughter)	155	12.9
Because I was influenced by a celebrity who follows the vegetarian diet	94	7.9
Because my friends are vegetarians	50	4.2
Because vegetarian products are more available in the environment than animal products	37	3.1
Because plant products are less expensive than nonplant products	34	2.8
Because someone in my family is following a vegetarian diet	29	2.4
Religious reasons	26	2.2
Other	5	0.4

### Sources of information about vegetarianism among vegetarian participants

Vegetarian participants relied on different sources of information about their diet. A total of 751 (62.7%) of the vegetarian participants depended on social media, 476 (39.8%) obtained information from vegetarian dietitians on social media, 450 (37.6%) relied on online sources (Wikipedia, journals, and forms), and 399 (33.3%) relied on scientific research. There were 153 participants (12.8%) who reported getting information from social media influencers and 5 (0.4%) who reported that they relied on YouTube.

### Challenges vegetarians face in maintaining a vegetarian diet

There were many challenges that influenced the participants’ adherence to a vegetarian diet. The top challenge they faced was the difficulty in finding meat and dairy substitutes, followed by the culture of the surrounding society; these challenges were reported by 209 (17.5%) and 178 (14.9%) of the participants, respectively. The unavailability of vegetarian dishes at parties and social events was common among 160 participants; 156 (13%) participants reported laziness and a lack of cooking skills, while 140 (11.7%) reported that they did not know what to eat, 136 (11.4%) mentioned the high cost, 133 (11.1%) reported the lack of vegetarian dishes in cafes and restaurants, 84 (7%) reported peer pressure, 76 (6.3%) reported a lack of diversity in their diet, 51 (4.3%) reported a fear of nutrient deficiencies, 28 (2.3%) reported missing meat and meat products, 16 (1.3%) reported food allergies, and 11 (0.9%) reported health problems. Thirty (2.5%) participants reported that they did not have challenges.

When asked about the ease of following a vegetarian diet, 611 (51%) of the vegetarian participants responded that it was generally easy.

### Beliefs and knowledge about vegetarianism among vegetarian and non-vegetarian participants

Knowledge of vegetarianism was measured by asking a set of questions, as shown in Table 4. Participants who correctly answered two questions or less were classified as having a low knowledge level. Participants who correctly answered more than two questions were classified as having a moderate to high knowledge level. Using these criteria, 92.9% of vegetarian participants were considered to have a low knowledge level.

**Table 4:** Beliefs and knowledge about vegetarianism among vegetarian participants (N = 1,197)

Beliefs Section			
	Agree n (%)	Disagree n (%)	I do not know n (%)
**Vegetarian products are more expensive than nonvegetarian products**	129 (10.8)	418 (34.9)	650 (54.3)
**A vegetarian diet is healthy**	95 (7.9)	54 (4.5)	1,048 (87.6)
**It is hard for a vegetarian diet to be balanced**	144 (12)	783 (65.4)	270 (22.6)
**Nonvegetarian products are more available than vegetarian products**	138 (11.5)	709 (59.2)	350 (29.2)
**Knowledge Section**			
**Vegetarians need supplements**	175 (14.6)	702 (58.6)	320 (26.7)
**A vegetarian diet does not have enough protein**	94 (7.9)	1,009 (84.3)	94 (7.9)
**Vegetarians are more vulnerable to deficiencies in vitamins and minerals such as vitamin B12, iron, and calcium**	195 (16.3)	720 (60.2)	282 (23.6)
**Fermented soya products are a good source of vitamin B12**	621 (51.9)	103 (8.6)	473 (39.5)

Table 5 shows the beliefs, perceptions and knowledge of vegetarianism among nonvegetarian participants. Participants who correctly answered two questions or less were classified as having a low knowledge level. Participants who correctly answered more than two questions were classified as having a moderate to high knowledge level. Using these criteria, 1182 (64.30%) had low knowledge levels, and 656 (35.7%) had moderate to high knowledge levels.

**Table 5:** Beliefs and knowledge about vegetarianism among nonvegetarian participants (n = 1838)

Beliefs Section			
	Agree n (%)	Disagree n (%)	I do not know n (%)
**The vegetarian diet is healthy.**	308 (16.8)	683 (37.2)	847 (46.1)
**It is hard for a vegetarian diet to be balanced**	340 (18.5)	411 (22.4)	1087 (59.1)
**Nonvegetarian products are more available than vegetarian products.**	359 (19.5)	882 (48)	597 (32.5)
**Knowledge Section**			
**Vegetarians need supplements**	379 (20.6)	305 (16.6)	1154 (62.8)
**All vegetarian diets do not have enough protein**	381 (20.7)	777 (42.3)	680 (37)
**Vegetarians are more vulnerable to have vitamin and mineral deficiencies, such as vitamin B**_**12**_, **iron and calcium**	583 (31.7)	312 (17)	943 (51.3)
**Fermented soya products are a good source of vitamin B**_**12**_	1280 (69.6)	133 (7.2)	425 (23.1)
**Perception Section**			
	Yes n (%)	No n (%)	
**Would you like to incorporate more vegetarian food in your diet?**	1507 (82)	331 (18)	

**Table 6:** Association Between Socio-Demographic Variables and Being Vegetarian (N = 3,035)

Demographic Characteristics	Are you Vegetarian?		*P*-Value
	Yes n = 1,197	No n = 1,838	
**Gender**			< 0.001**
Female	1,079 (44.3%)	1,355 (55.7%)	
Male	118 (19.6%)	483 (80.4%)	
**Age**			< 0.001**
18–39	1,158 (41.8%)	1,614 (58.2%)	
40–50	30 (15.6%)	162 (84.4%)	
51–64	5 (7.7%)	60 (92.3%)	
65 years and older	4 (66.7%)	2 (33.3%)	
**Nationality**			0.04*
Saudi	1,138 (39.9%)	1,714 (60.1%)	
Non-Saudi	59 (32.2%)	124 (67.8%)	
**Place of Residency**			< 0.001**
Makkah Region	364 (33.1%)	735 (66.9%)	
Madinah Region	85 (38.6%)	135 (61.4%)	
Riyadh Region	321 (42.6%)	433 (57.4%)	
Qasim Region	79 (48.8%)	83 (51.2%)	
Baha Region	48 (48%)	52 (52%)	
Tabuk Region	20 (42.6%)	27 (57.4%)	
Northern Boarders Region	23 (43.4%)	30 (56.6%)	
Eastern Region	181 (44.1%)	229 (55.9%)	
Jouf Region	21 (70%)	9 (30%)	
Jazan Region	13 (26%)	37 (74%)	
Najran Region	6 (42.9%)	8 (57.1%)	
Other	36 (37.5%)	60 (62.5%)	
**Marital Status**			< 0.001**
Single	1,017 (47.8%)	1,110 (52.2%)	
Engaged	22 (30.6%)	50 (69.4%)	
Married	125 (16.8%)	618 (83.2%)	
Widowed	5 (50%)	5 (50%)	
Divorced	24 (33.8%)	47 (66.2%)	
Separated	4 (33.3%)	8 (66.7%)	
**Educational Level**			< 0.001**
Intermediate or less	24 (42.1%)	33 (57.9%)	
High school	395 (48.6%)	418 (51.4%)	
Diploma	56 (34.8%)	105 (65.2%)	
Bachelor’s degree	659 (39.1%)	1,027 (60.9%)	
Higher Education	63 (19.8%)	255 (80.2%)	
**Job**			< 0.001**
In the public sector	51 (19.4%)	212 (80.6%)	
In the health sector	52 (19%)	222 (81%)	
In the health sector but not a health practitioner	13 (31%)	29 (69%)	
In the private sector	90 (31.4%)	197 (68.6%)	
In the army / police	7 (15.9%)	37 (84.1%)	
Self-employed	39 (42.9%)	52 (57.1%)	
I do not work	347 (44.8%)	427 (55.2%)	
Housewife	24 (14.7%)	139 (85.3%)	
Student	560 (53.2%)	493 (46.8%)	
Retired	8 (25.8%)	23 (74.2%)	
Unable to work	6 (46.2%)	7 (53.8%)	
**Income**			< 0.001**
Less than 5,000 SR/month	982 (46.9%)	1,113 (53.1%)	
5,000–10,000 SR/month	101 (24.1%)	318 (75.9%)	
10,000–15,000 SR/month	60 (22.7%)	204 (77.3%)	
15,000–20,000 SR/Month	25 (20.5%)	97 (79.5%)	
More than 20,000 SR/month	29 (21.5%)	106 (78.5%)	
**BMI**			< 0.001**
Underweight	97 (49.7%)	98 (50.3%)	
Normal BMI	821 (46.9%)	930 (53.1%)	
Overweight	173 (27.1%)	465 (72.9%)	
Obesity 1	64 (25%)	192 (75%)	
Obesity 2	21 (15.9%)	111 (84.1%)	
Obesity 3	21 (33.3%)	42 (66.7%)	
**Do you smoke?**			< 0.001**
Yes	55 (21.4%)	202 (78.6%)	
No	1,032 (40.7%)	1,502 (59.3%)	
Occasionally	110 (45.1%)	134 (54.9%)	
**If you exercise, how much time do you usually spend daily?**			< 0.001**
I do not exercise	371 (29.5%)	885 (70.5%)	
Half an hour	388 (41.8%)	541 (58.2%)	
An hour	314 (49.5%)	320 (50.5%)	
Two hours	105 (60.7%)	68 (39.3%)	
More than two hours	19 (44.2%)	24 (55.8%)	

* Significant at level ***P*** **≤ 0.05**

** Significant at level ***P*** **≤ 0.005**

### Factors associated with being vegetarian

There was a significant association between being a vegetarian and sex, age, residence, marital status, educational level, job, income, BMI, smoking and exercise habits (all *P* < 0.005) and nationality (*P* < 0.05). Vegetarians were more likely to be female, young, residing in the Al-Jouf region, single, holding high school certification, students, exercising more than two hours a day and Saudis (Table 6).

### Association between vegetarianism and chronic disease

A significant difference was observed between vegetarians and nonvegetarians in the prevalence of diseases. Vegetarians had a higher incidence of depression (*P* ˂ 0.001) and anxiety (*P* ˂ 0.001) than nonvegetarians. Nonvegetarians had a significantly higher prevalence of anaemia (*P* ˂ 0.001), thyroid disorders (*P* = 0.002), diabetes (*P* = 0.033), CVD and hypertension (*P* = 0.017), and abnormal lipid profiles (*P* ˂ 0.001) than vegetarians.

### Association between knowledge level and demographic variables among vegetarians

There was no association between age (*P* = 0.49), educational level (P = 0.31), or presence of disease (P = 0.5) and the knowledge level of vegetarians.

### Likelihood of being vegetarian

A multivariate logistic regression included the following variables: sex, age, marital status, educational level, job, income, smoking habits, presence of chronic diseases, and exercise habits. Participants with the following characteristics were significantly (P < 0.05) more likely to be vegetarian: exercising for half an hour, exercising for an hour, and exercising for two hours. Presence of the following characteristics was significantly (P < 0.05) correlated with lower chances of being a vegetarian: male, older age of 51–64 years, being married, having a higher educational level, working in the health sector, being a housewife, and having a medium income between 5,000 and 10,000 SR/month (approximately equals 1333–2665 USD), as shown in Table 7.

**Table 7:** Factors that Predict Being Vegetarian

Demographics	*P*-Value	Odds Ratio	Confidence Interval
**Gender (Female vs. Male)**	< 0.001**	0.336	0.259–0.434
**Age (18**–**39 is the Referent)**			
40–50	0.062	0.641	0.401–1.022
51–64	0.004**	0.204	0.068–0.61
65 years and older	0.16	4.712	0.541–41.03
**Marital Status (Single is the Referent)**			
Engaged	0.087	0.621	0.36–1.072
Married	< 0.001**	0.45	0.341–0.592
Widowed	0.366	2.055	0.431–9.792
Divorced	0.589	0.858	0.491–1.497
Separated	0.673	1.323	0.361–4.85
**Educational Level (Intermediate or less is the Referent)**			
High school	0.702	0.89	0.489–1.618
Diploma	0.583	0.825	0.415–1.641
Bachelor’s Degree	0.376	0.766	0.424–1.384
Higher Education	0.011**	0.419	0.214–0.819
**Job (In the public sector is the Referent)**			
In the health sector	0.012*	0.542	0.337–0.872
In the health sector but not a health practitioner	0.748	1.14	0.512–2.538
In the private sector	0.401	1.213	0.773–1.905
In the army / police	0.795	0.884	0.347–2.247
Self-employed	0.266	1.398	0.775–2.52
I do not work	0.743	1.077	0.691–1.678
Housewife	0.028*	0.507	0.277–0.931
Student	0.138	1.397	0.898–2.174
Retired	0.063	2.864	0.944–8.687
Unable to work	0.765	1.211	0.346–4.244
**Income (Less than 5000 SR/month is the Referent)**			
5,000–10,000 SR/month	0.003**	0.637	0.472–0.86
10,000–15,000 SR/month	0.477	0.87	0.593–1.276
15,000–20,000 SR/month	0.987	1.005	0.588–1.716
More than 20,000 SR/month	0.712	1.102	0.657–1.85
**Do you smoke? (Yes is the Referent)**			
No	0.617	1.096	0.766–1.569
Occasionally	0.026	1.647	1.062–2.556
**Do you have any disease? (No vs. Yes)**	0.745	1.028	0.872–1.211
**If you exercise, how much time do you usually spend daily? (I do not exercise is the Referent)**			
Half an hour	< 0.001**	1.714	1.414–2.077
An hour	< 0.001**	2.321	1.872–2.877
Two hours	< 0.001**	2.321	2.742–5.662
More than two hours	0.202	1.543	0.793–3.003

* Significant at level ***P*** **≤ 0.05**

** Significant at level ***P*** **≤ 0.005**

## Discussion

To our knowledge, this is the first study to investigate knowledge and attitudes about vegetarianism and associated factors in the Saudi population and to understand the reasons for its growing prevalence. Despite the strong influence of a tradition of meat-based consumption, findings from this study prove that there is a significant number of vegetarians and vegans in Saudi Arabia. The prevalence of self-reported vegetarianism among the general Saudi population found in the current study was 37.5%. This study does not claim that this is an accurate figure for the prevalence of vegetarianism in the country; it is to be expected that people with an interest in vegetarianism would self-select for participation [24]. Comments indicated that participants were pleased to be asked about their diet, as this was the first opportunity that they had to share their views.

The majority of our sample was female, which might be due to many factors. Female individuals tend to care more about food, body shape and weight loss [25–28]. Additionally, women in Saudi Arabia are more likely to answer online surveys and to spend more time on social media [28]. It is possible that the recruitment process (through social media) resulted in an overrepresentation of young people. However, this number may be due to an increased awareness of the health risks of consuming large amounts of meat. Health reasons were observed among the strongest motives for adopting a vegetarian diet among Saudis, whereas Heiss, Hormes and Alix Timko (2017) found that moral motives were most prominent in Western societies. Studying vegetarianism is challenging for researchers due to the difficulty in identifying vegetarians, differentiating between types of vegetarianism, and interpreting the discrepancy between self-reported identification and self-reported behaviour [10, 27, 29]. Nonvegetarians have low levels of knowledge and understanding of a vegetarian diet. This might be due to the lack of understanding and awareness of vegetarianism in Saudi Arabia. A recent Saudi study conducted by AlHusseini and his colleagues confirmed the previous suggestion that knowledge about vegetarianism in Saudi individuals was fair [24]. Moreover, it has been reported that vegetarians are more likely to develop nutritional deficiencies due to a lack of knowledge [29, 30].

The current study found interesting results regarding vegetarians’ beliefs about vegetarianism. Vegetarians indicated that vegetarian products are easily available and are not expensive. This may also help to explain the spread of vegetarianism in Saudi Arabia. Some studies have indicated that vegetarian products are often described as expensive in Western societies, which reduces the chance of people adopting the diet [30, 31]. On the other hand, Lusk and Norwood (2016) found that vegetarians reported lower food expenditures than meat eaters. While other studies have found that most vegetarians believe that a vegetarian diet is healthy, a neutral attitude was observed of vegetarians regarding this belief in the current study [32–34]. Most studies indicated that they did not know whether this belief was valid [32–34]. However, vegans were more likely to have good health and to lead a healthy lifestyle, including regular exercise, good sleeping patterns, better gut microbiota, lower gut inflammation, lower blood pressure, and a reduced total HDL cholesterol ratio [33].

In general, the knowledge level of vegetarians was low, contrary to expectations. Although many studies have found that vegetarians with a higher educational level have a higher level of knowledge about vegetarianism [32–34], this relationship was not found in the current study or in some others [35, 36].

There is no doubt that there are social and psychological factors that affect an individual’s dietary behaviour, preferences, and taste. These sociopsychological influences may either support or inhibit a healthy lifestyle [37, 38]. In the current study, vegetarians indicated that social norms constitute a barrier to adherence to a vegetarian diet. These social norms include the prevailing food culture (consumption of meat) on special occasions, holidays, and family gatherings and showing generosity by providing meat on these special occasions. Moreover, refusing to eat what is given is considered impolite in Saudi culture. Because of this, there may be many Saudis who are open to vegetarianism but who find it difficult to adopt a vegetarian diet while surrounded by these rigid traditions. It has been shown that most omnivores describe vegetarians as virtuous but weak, more moral, and less masculine [36]. In a survey conducted by Rosenfeld and Tomiyama (2020), many meat eaters indicated that the fear of feeling stigmatized was one of the main barriers that prevented them from adopting vegetarianism. Financial cost, sensory enjoyment of meat, inadequate cooking skills, and convenience (lack of vegetarian dish variety) were also reported as barriers in this study as well as other studies [40–42].

Today, with technology, it is easy to access detailed information on any topic and gain access to many cultures around the world. Accordingly, social networks have a clear effect on an individual’s behaviours, especially dietary behaviours [43, 44]. In the current study, 62.7% of vegetarians reported relying on social media such as Twitter, Facebook, and Instagram to gain information about vegetarianism. Social media can be a useful tool for facilitating dietary and health knowledge. However, not all influencers and accounts promote accurate health information.

The current study found many factors that had a statistically significant association with choosing a vegetarian lifestyle, including sex, age, income level, educational level, place of residence, BMI, marital status, job, and some health-related behaviours such as smoking and exercise habits. Men were less likely to be vegetarian than women. Many studies have found that most vegetarians are young women [25–27]. It has been observed that women are more likely than men to adopt a vegetarian diet for weight loss and body image reasons [39]. Additionally, in Saudi Arabia, feasting and gathering are gender segregated, and vegetarian food is widely available for women. Typically, men are served meat-based dishes, whereas women are able to choose from a buffet.

It was observed that with increasing age, it is less likely to change certain behaviours, including dietary patterns. The current study found that people aged 51–64 were less likely to be vegetarians than those aged 18–39, which is consistent with other studies that have found that more younger adults tend to adopt a vegetarian lifestyle than older adults [26, 45]. A study conducted in the UK researched attitudes concerning different reasons to be vegetarian across generations. The results showed that younger people [11–20] were broadly in agreement with moral and environmental reasons. People aged 41–60 agreed with health reasons [41]. Although many studies have indicated that vegetarians have higher educational levels than meat eaters [25, 26, 45, 46], the current study found a different result; those with a higher educational level were less likely to be vegetarians. The explanation for this difference may be due to the explosion in online knowledge. The current study found that those with an income of between 5,000 and 10,000 SR were less likely to be vegetarians than those with an income of less than 5,000 SR. The prevalence of vegetarianism may vary according to income level. Rammohan et al. (2012) indicated that poverty and the high cost of meat were reasons for choosing a vegetarian diet, while some other studies have indicated that people with a higher income are more likely to be vegetarians for health and environmental reasons [47, 48].

It is possible that people who are health conscious in general are more likely to exercise, avoid smoking, and adopt a vegetarian diet. The current study found that people who spent two hours exercising a day were 3.94 times more likely to be vegetarians than those who did not exercise. Several studies have indicated that exercising and avoiding smoking are closely associated with vegetarianism [49, 50].

Regarding the potential risks or benefits of a vegetarian diet for mental health, the current study found significant associations between vegetarianism, depression, and anxiety, which is consistent with a number of previous studies [52–53]. On the other hand, a recent systematic review and meta-analysis of observational studies concluded that there were no significant associations between a vegetarian diet and depression [45]. The results are still contradictory as to whether a vegetarian diet is associated with positive or negative effects on mental health. For example,Jin et al. (2019) concluded that vegetarianism was inversely related to the prevalence of depression in middle-aged South Asians in America.Beezhold et al. (2015) conducted an online survey to investigate the moods of adult vegetarians and meat eaters, and they concluded that male vegans were less anxious than male vegetarians and omnivores, while female vegans were less stressed than female vegetarians and omnivores. In contrast to these studies, a study conducted on university students in the USA showed that vegetarians were more likely to suffer from neuroticism and depression than omnivores [47]. A study found that the risk of depression among vegetarian men was 1.67 times higher than the risk for nonvegetarians [48]. This might be due to an increased risk of deficiencies in certain nutrients, such as iron (64–66), zinc (67,68), omega-3 (69–72), vitamin B_12_ (73–75), and vitamin D (76–78). These nutrients are mostly found in animal dietary sources.

A significant association was found between CVD, hypertension, diabetes, abnormal lipid profiles and not being a vegetarian. A vegetarian diet has been associated with a reduced risk of some chronic diseases, such as hypertension, diabetes, and CVD (79–82). It has been shown that vegetarians have lower levels of total cholesterol, triglycerides, fasting glucose, and diastolic and systolic blood pressure than nonvegetarians [54]. Vegetarianism is also inversely associated with fasting glucose concentrations, insulin resistance, total and LDL cholesterol concentrations, visceral fat levels, and fatty liver prevalence [55]. The current study found a significant association between anaemia and nonvegetarian status. This might be due to the large proportion of women in the study, and women are more likely than men to have anaemia.

There are some limitations of the current study to consider. Short-term vegetarians were included in the survey. The current study identified people who had adhered to a vegetarian diet for a month or less as being vegetarians. Another limitation was that dietary habits were self-reported; it is therefore difficult to know if these data are entirely accurate. Previous studies have shown that self-reported vegetarians do not always completely abstain from meat. No conclusions can be drawn regarding the causal relationship between diet and the individual variables examined because the study was a cross-sectional design. Additionally, the majority of the participants were female. We did not investigate the use of vitamins and supplements among the participants. Conducting an online survey instead of face-to-face or phone interviews can also be a source of bias for the study since it limits the results to individuals who have internet access. On the other hand, the current study has many strengths. This study is the first to examine the prevalence of vegetarianism in Saudi Arabia and to examine knowledge and perceptions of vegetarianism in the country. The sample size was large and can be considered representative of the general Saudi population. It would be interesting to follow-up with participants in a cohort study.

## Conclusion

To conclude, approximately one-third of the participants were vegetarians. Almost all the vegetarian participants had a low knowledge level regarding vegetarianism. Health issues, followed by ethical and environmental concerns, were the most common motivators for adopting a vegetarian diet. Factors associated with increased vegetarianism were having daily exercise habits of less than two hours, whereas factors associated with reduced vegetarianism were male, older age, married people, high educational level, working in the health sector, being a housewife, and medium monthly income. This study can open doors for more research to be done in the field of vegetarianism. A nationwide study of dietary preferences should be conducted to estimate the actual number of vegetarians. The level of knowledge among our participants was slightly low, and this needs governmental effort to improve their knowledge though the Ministry of Health rather than relying on unofficial sources. Educational programmes should be carried out to improve knowledge about different diets and how to achieve optimal nutrition.

## Data Availability

The raw data supporting the conclusions of this article will be made available by the authors without undue reservation. Please contact Dr. Khloud Ghafouri. Kjghafouri@uqu.edu.sa.
